# Clinical and Genetic Determinants of Warfarin Pharmacokinetics and Pharmacodynamics during Treatment Initiation

**DOI:** 10.1371/journal.pone.0027808

**Published:** 2011-11-16

**Authors:** Inna Y. Gong, Ute I. Schwarz, Natalie Crown, George K. Dresser, Alejandro Lazo-Langner, GuangYong Zou, Dan M. Roden, C. Michael Stein, Marc Rodger, Philip S. Wells, Richard B. Kim, Rommel G. Tirona

**Affiliations:** 1 Department of Physiology & Pharmacology, Schulich School of Medicine and Dentistry, University of Western Ontario, London, Ontario, Canada; 2 Division of Clinical Pharmacology, Department of Medicine, Schulich School of Medicine and Dentistry, University of Western Ontario, London, Ontario, Canada; 3 Division of Hematology, Department of Medicine, Schulich School of Medicine and Dentistry, University of Western Ontario, London, Ontario, Canada; 4 Department of Epidemiology and Biostatistics, Robarts Clinical Trials, Schulich School of Medicine and Dentistry, University of Western Ontario, London, Ontario, Canada; 5 Division of Clinical Pharmacology, Departments of Medicine and Pharmacology, Vanderbilt University Medical Center, Nashville, Tennessee, United States of America; 6 Department of Medicine, University of Ottawa, Ottawa Health Research Institute, Ottawa, Ontario, Canada; Dr. Margarete Fischer-Bosch Institute of Clinical Pharmacology, Germany

## Abstract

Variable warfarin response during treatment initiation poses a significant challenge to providing optimal anticoagulation therapy. We investigated the determinants of initial warfarin response in a cohort of 167 patients. During the first nine days of treatment with pharmacogenetics-guided dosing, *S*-warfarin plasma levels and international normalized ratio were obtained to serve as inputs to a pharmacokinetic-pharmacodynamic (PK-PD) model. Individual PK (*S*-warfarin clearance) and PD (I_max_) parameter values were estimated. Regression analysis demonstrated that *CYP2C9* genotype, kidney function, and gender were independent determinants of *S*-warfarin clearance. The values for I_max_ were dependent on *VKORC1* and *CYP4F2* genotypes, vitamin K status (as measured by plasma concentrations of proteins induced by vitamin K absence, PIVKA-II) and weight. Importantly, indication for warfarin was a major independent determinant of I_max_ during initiation, where PD sensitivity was greater in atrial fibrillation than venous thromboembolism. To demonstrate the utility of the global PK-PD model, we compared the predicted initial anticoagulation responses with previously established warfarin dosing algorithms. These insights and modeling approaches have application to personalized warfarin therapy.

## Introduction

The vitamin K antagonist, warfarin, is an oral anticoagulant commonly prescribed to prevent and treat venous thromboembolism (VTE) and decrease the risk of stroke in atrial fibrillation (AF).[1] Warfarin therapy is complicated by the wide interindividual variation in response and dose requirements for adequate anticoagulation. Optimal warfarin therapy is achieved by maintaining the anticoagulation response, international normalized ratio (INR), within a narrow therapeutic range of 2.0 to 3.0 for most indications. Due to the unpredictable pharmacokinetic (PK) and pharmacodynamic (PD) responses to warfarin, initiation of therapy is the most clinically challenging phase as the optimal dose is often determined iteratively, guided by INR.[2]

Warfarin is administered as a racemic drug; however, the *S*-warfarin enantiomer is 3–5 times more potent than *R*-warfarin.[3] *CYP2C9* is the primary enzyme responsible for metabolism of *S*-warfarin,[4] and studies have consistently shown that *CYP2C9* polymorphisms (^*^2, c.430C>T, rs1799853; ^*^3, c.1075A>C, rs1057910) significantly contribute to the variable warfarin response.[5] Non-genetic factors of warfarin PK variability and dose requirements are also important. For example, age and co-administration with drugs that inhibit or induce *CYP2C9* can alter *S*-warfarin elimination.[6], [7], [8], [9], [10] Moreover, *S*-warfarin volume of distribution is dependent on weight.[11], [12] Taken together, it has been estimated that PK factors determine 26-40% of warfarin maintenance dose variability.[10], [13], [14]

Warfarin exerts its anticoagulation effects by inhibiting vitamin K epoxide reductase (VKOR encoded by the *VKORC1* gene), the enzyme responsible for recycling oxidized vitamin K epoxide to its hydroquinone form, an essential cofactor for activation of clotting factors II, VII, IX and X.[15] It is appreciated that single nucleotide polymorphisms (SNPs) in *VKORC1* result in altered warfarin sensitivity while rare mutations have been linked to warfarin resistance.[8], [16] Of note, the common promoter SNP (*VKORC1* -1639G>A, rs9923231) is likely the causative variation responsible for greater warfarin sensitivity.[17], [18] In addition to *CYP2C9* and *VKORC1* polymorphisms, several studies have reported that a functional SNP in *CYP4F2* (c.1297G>A, rs2108622), the metabolizing enzyme for vitamin K,[19] also determines dose requirement.[20], [21] Furthermore, diet has long been considered an important environmental determinant of warfarin response. Indeed, reduced anticoagulation response was observed in warfarin-stabilized patients with intake of vitamin K-rich foods,[22], [23] and vitamin K status was associated with warfarin sensitivity at the onset of treatment.[24]

With the intent of improving warfarin anticoagulation therapy, a number of algorithms have been proposed which incorporate genetics as well as clinical parameters to predict individualized maintenance dose.[8], [25], [26] Many of the factors influencing required maintenance dose such as age, body surface area, drug interactions and importantly, *CYP2C9* genotype relate to their effects on *S*-warfarin PK parameters, such as volume of distribution and clearance.[7], [8], [9], [27] The influence of genetics and clinical parameters on *S*-warfarin PD variability is less clear. Although the influence of *VKORC1* genetic variations and vitamin K intake on dose and anticoagulation response is evident, the quantitative and dynamic influence of these variables on PD parameters, such as drug affinity and maximal inhibition, has not been well established.[28] Moreover, there is a paucity of information regarding the influence of other genetic and clinical variables on *S*-warfarin PD variation.

In this study, we aimed to separate warfarin pharmacokinetic factors from intrinsic pharmacodynamic factors to elucidate crucial covariates of each, and their contribution to the overall anticoagulation response variation. To this end, PK-PD modeling was applied to a cohort of patients commencing warfarin therapy using a novel initiation protocol.[29]

## Materials and Methods

### Study subjects and design

Patients with AF (n = 61), VTE (n = 98) or other conditions (n = 8) were prospectively enrolled to evaluate the safety and efficacy of a pharmacogenetics-based warfarin initiation protocol. Patient characteristics and clinical outcomes were described previously in detail. [29] The inclusion criteria for study enrolment were minimum of 18 years of age and indication for new warfarin therapy for at least 3 months with a target INR range of 2.0 to 3.0. Patients were excluded on the basis of diagnosis of cancer other than non-melanoma skin cancer, alcohol or drug abuse, baseline INR>1.4, known warfarin allergy/intolerance, terminal disease, prior use of warfarin or vitamin K use within 2 weeks prior to study enrolment, and pregnancy. The majority of patients were Caucasian (95%) with mean age of 60 years (range, 19–88) and mean weight of 84 Kg (43–155). The allelic frequencies for *VKORC1* -1639G>A and *CYP4F2* c.1297G>A were 38.0% and 31.7%, respectively. The *CYP2C9* ^*^2 and ^*^3 allelic frequencies were 11.1% and 4.8%, respectively. There was no homozygous *CYP2C9* ^*^3 carrier in this population. Amiodarone, statin, antiplatelet, antibiotic, antifungal and NSAID medication use were present in 2%, 45%, 55%, 6%, 1% and 12% of the cohort, respectively.

The Warfarin Regimen using A Pharmacogenetics-guided Initiation Dosing (WRAPID) protocol has been described elsewhere.[29] Briefly, a 2-day loading dose (according to *VKORC1* and *CYP2C9* genotype) was administered, followed by a day 3 INR measurement that was used in combination with the maintenance algorithm to determine the subsequent dose. Two subsequent INR measurements were obtained within the first 9 days of therapy where the maintenance dose was further adjusted accordingly to the dose adjustment nomogram. Simultaneous with INR monitoring, additional blood samples were collected for drug level analysis.

This study was conducted at the London Health Sciences Centre and The Ottawa Hospital upon approval by Research Ethics Boards at the University of Western Ontario and Ottawa Hospital. Patients requiring initiation of warfarin therapy were prospectively screened for study eligibility and informed written consent was acquired.

### Genotyping

Genomic DNA was isolated with Gentra Puregene or DNA Blood Midi extraction kit (Qiagen, Valencia, CA). At London Health Sciences Centre, genotypes were determined by allelic discrimination using TaqMan Drug Metabolism Genotyping assays with the 7500 RT-PCR System (Applied Biosystems, Carlsbad, CA). At Ottawa Health Research Institute, genotypes were determined using the Luminex 200 system (Luminex, Austin, TX).

### Warfarin drug level analysis

Racemic warfarin and internal standard (IS) *R/S*-*para*-chloro-warfarin were purchased from Sigma-Aldrich. Plasma was extracted from patient blood samples within 1 hour of collection and stored at −80°C until use. Total *S-*warfarin plasma concentration was determined using liquid chromatography-tandem mass spectrometry (LC-MS/MS). Briefly, 300 µL of acetonitrile and 25 µL of IS was added to 100 µL of plasma and centrifuged at 14,000 rpm for 20 min. The resulting supernatant was added to 5 mM ammonium acetate pH 4 (1∶3 v/v). Warfarin and IS enantiomers were separated with the Astec CHIROBIOTIC™ V Chiral Column (5 cm×4.6 mm, 5 µM particle size) using gradient elution with 5 mM ammonium acetate (pH 4) and acetonitrile (5 to 70%) in a 10 min run time. The MS was set in negative mode for detection of warfarin and IS with transitions 307.2 160.0 *m/z* and 340.8 160.0 *m/z*, respectively. Calibration curves were prepared by spiking blank plasma with known concentrations of *R/S*-warfarin. The lowest limit of quantification was 1 ng/mL for both enantiomers. The interday coefficient of variation and bias of *S*-warfarin quality controls was 10.5% and 9.3%.

### Proteins induced by vitamin K absence factor II (PIVKA-II) assay

PIVKA-II concentrations were analyzed with use of an enzyme-linked immunosorbent assay kit as per manufacturer's protocols (Diagnostica-Stago, Parsippany, NJ).

### Kidney function

We measured patient plasma creatinine concentrations by LC-MS/MS. Briefly, creatinine and the IS, creatinine-D3, was purchased from Sigma-Aldrich and Toronto Research Chemicals, respectively. Creatinine and IS were separated with the reverse-phase Hypersil Gold column (50×5 mm, 5 µM particle size) using isocratic elution with 25% 1% formic acid in water v/v and 75% acetonitrile with 1% formic acid v/v in a 7 min run time. The MS was set in positive mode for detection of creatinine and IS with transitions 114.1 → 44.3 *m/z* and 117.1 → 47.3 *m/z*, respectively. The lowest limit of quantification was 50 ng/mL. The interday coefficient of variation and bias of creatinine quality controls was 8.7% and 6%. eGFR was estimated using the Chronic Kidney Disease Epidemiology Collaboration (CKD-EPI) equation.[30] Renal function was categorized according the National Kidney Foundation's classification of chronic kidney disease.

### PK-PD modeling

*S*-warfarin PK was described using a linear one-compartment model with a set volume of distribution (V; 0.14 L/kg) on a per patient basis.[12] The time-course of plasma *S*-warfarin concentration (C_p_) arose from the interplay between first-order drug absorption (k_a_) and drug elimination (k_e_) processes. Parameter values for k_a_ were fixed (28.56 day^−1^) based on the literature.[31] Bioavailability was assumed to be complete.[32] Individual k_e_ values were obtained by least squares fitting (Scientist, Micromath, St. Louis, MO) of the concentration data during the first 9 days with prescribed doses as input. Clearance (CL) was calculated as the product of V and k_e_.

*S*-warfarin PD was described by an established indirect response model which incorporates the known delay in anticoagulation effects.[33] In this model, the rate of change in INR was modeled using zero-order input (K) and first-order output (k_out_) variables. Plasma *S*-warfarin levels (C_p_) modulate the output response according to classical inhibition kinetics, described by parameters maximum inhibitory factor (I_max_, i.e. inversely related to enzyme content) and drug affinity (IC_50_).[34] Since *VKORC1* -1639G>A promoter SNP has been correlated with altered mRNA expression levels, I_max_ values were expected to vary with *VKORC1* genotype. R_max_ and k_out_ values in the indirect response model were both fixed at 1. The IC_50_ for *S*-warfarin was fixed at 1500 ng/mL, as reported previously.[35] The following equation describes the PD model.
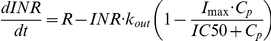
The response analysis was conducted following estimation of individual *S*-warfarin plasma concentrations. These estimated drug concentrations were used in combination with measured INRs to estimate the individual PD parameter, I_max_, by least squares fitting.

We note that clearance and I_max_ parameter estimates should be considered independent of the dosing regimen and anticoagulation responses observed in the WRAPID study because estimations of individual warfarin clearance and individual drug concentration-response profile are unaffected by the doses received.

### Vitamin K epoxide reductase protein expression in human liver

The collection and processing of liver samples was described elsewhere.[36] In order to obtain a positive control for VKOR protein analysis, the enzyme was over-expressed in cells using previously described protocol.[37] For this purpose, human *VKORC1* cDNA was amplified from a human liver cDNA library using primers 5′-TGGAGATAATGGGCAGCACCTGGGGG-3′ (forward) and 5′-GTTGAGGGCTCAGTGCCTCTTAGCCTTG-3′ (reverse). Samples were separated by SDS-PAGE on 4–10% gels (Invitrogen, Carlsbad, CA) and subsequently transferred onto nitrocellulose membranes. Blots were probed with a custom anti-VKOR antibody (kindly provided by Dr. Kathleen Berkner, Learner Research Institute, Cleveland Clinic [38]) and subsequently probed with anti-rabbit horseradish peroxidise-labeled secondary antibodies (Bio-Rad, Hercules, CA). The bands were detected using the BM Chemiluminescence Western Blotting Substrate (Roche, Indianapolis, IN) and KODAK ImageStation 4000 MM (Carestream, Rochester, NY). Protein expression levels were normalized to a wild-type *VKORC1* sample (HLM100), repeated on all blots.

### Determinants of warfarin kinetics and response

Regression analysis was performed to determine factors affecting *S*-warfarin clearance and I_max_. Since the distribution of both of these parameters in our patient population was skewed, square-root transformation was adopted to normalize the data. The variables age, gender, body weight, amiodarone use, other known interacting medications, indication for warfarin therapy, kidney function, vitamin K status as measured by PIVKA-II, *VKORC1* genotype, *CYP2C9* ^*^2 and ^*^3 genotype were considered as covariates for both *S*-warfarin clearance and I_max_. The covariates were added to the model according to the stepwise forward regression. A P-value<0.05 was considered as significant and the variable was subsequently entered into the equation; variables included with P-values>0.1 in subsequent models were removed. The models with significant covariates were then internally validated through bootstrapping. Bootstrapping was achieved by random sampling with replacements to obtain 1000 samples, allowing estimation of the standard error and the 95% confidence interval (CI) of parameter estimates. Potential collinearity between variables was assessed using condition indices and variance proportions.

The clearance and I_max_ regression equations were then integrated with the PK-PD model in order to predict and compare anticoagulation response profiles following initiation with various nomograms for typical warfarin patients.

### Statistical analysis

The Kruskal-Wallis one-way analysis of variance followed by Tukey's test for pairwise comparisons was employed for the following analysis: *S*-warfarin concentration differences with respect to *VKORC1* genotype, influence of *VKORC1* genotype on attainment of therapeutic INR and dose, effect of *VKORC1* genotype on liver protein expression, relationship between *S*-warfarin clearance and *CYP2C9* genotype, effect of kidney function on *S*-warfarin clearance, relationship between *VKORC1* genotype and I_max_. Mann-Whitney U's test was employed to examine gender effect on *S*-warfarin clearance and warfarin indication effect on I_max_. A two-tailed P-value of less than 0.05 was considered significant for all analyses. Statistical analysis was performed with the use of GraphPad Prism v.5.0 (GraphPad, La Jolla, CA) or SPSS v. 17.0 (SPSS, Chicago, IL).

## Results

### PK-PD model performance

**Figure 1:**
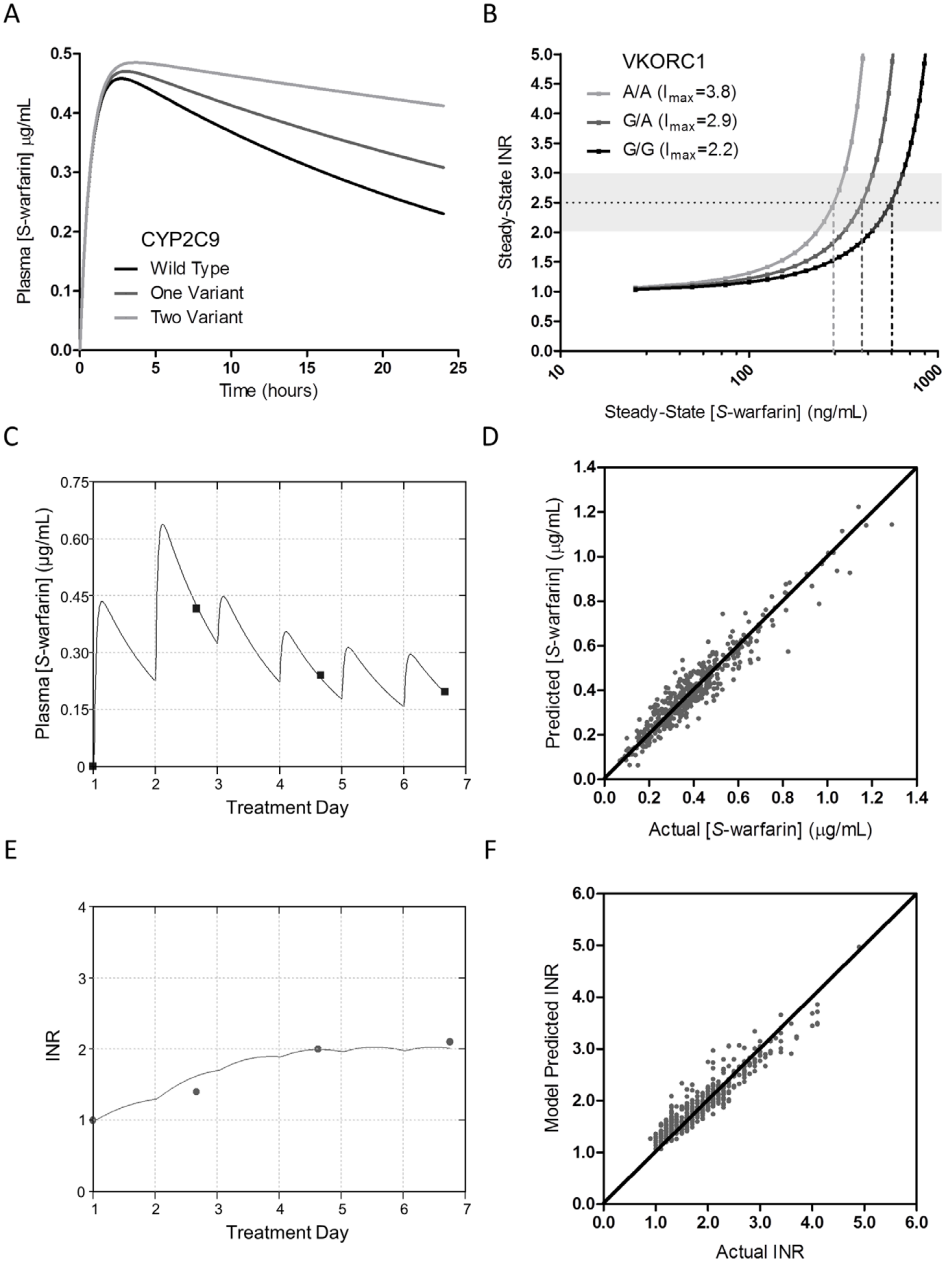
PK-PD model performance. (A) Model simulated *S*-warfarin plasma concentration-time profiles after single dose with *CYP2C9* variant alleles. (B) Model simulated steady-state therapeutic INR (2.5) *vs. S*-warfarin plasma concentration with varying I_max_ corresponding to *VKORC1* -1639G>A genotype. (C) Model fit of measured *S*-warfarin concentrations in a single patient. (D) Scatter plot of actual *vs.* predicted *S*-warfarin plasma concentration throughout the initiation phase (coefficient of determination, r^2^ = 0.91, n = 459). The diagonal line represents the unity line. (E) Model fit of measured anticoagulation INR response values in the same patient as in (C). (F) Scatter plot of actual *vs.* predicted INR during the initiation phase (r^2^ = 0.89, n = 459). The diagonal line represents the unity line. I_max_, maximal inhibitory factor; INR, international normalized ratio.

We fitted the individual patient *S*-warfarin plasma levels during the first 9 days of therapy to a one-compartment PK model (Figure 1A) to furnish estimates of *S*-warfarin clearance. The *S*-warfarin clearance estimated here was similar to that previously observed.[39] Moreover, good fits to individual patient levels with the PK model were obtained (Figure 1C). Overall, the PK model was sufficiently accurate in describing the data as linear regression analysis for predicted and actual *S*-warfarin concentrations yielded a coefficient of determination (r^2^) of 0.91, with a slope of 0.92 (Figure 1D). The mean absolute error (MAE) between estimated and actual was 0.04 µg/mL, and 88% of these estimated values were within 25% of actual concentrations.

An indirect response model was used to estimate maximal inhibitory factor (I_max_), the PD parameter related to the amount of hepatic VKOR enzyme. Here, the *S*-warfarin plasma concentration-INR response relationship is governed by the parameters IC_50_ (related to warfarin affinity to VKOR) and I_max_, where at constant IC_50_, increasing I_max_ enhances drug sensitivity (Figure 1B). Individual predicted *S*-warfarin concentrations estimated from the PK model in conjunction with observed INR values served as inputs for the PD model. Fits to individual patient INRs over the initiation period were good (Figure 1E). Linear regression analysis for predicted and actual INR values of the entire data set yielded an r^2^ of 0.89, with a slope of 0.91 (Figure 1F). The MAE was 0.17, and 90% of these estimated values were within 25% of actual INR.

### Determinants of *S*-warfarin clearance

**Figure 2:**
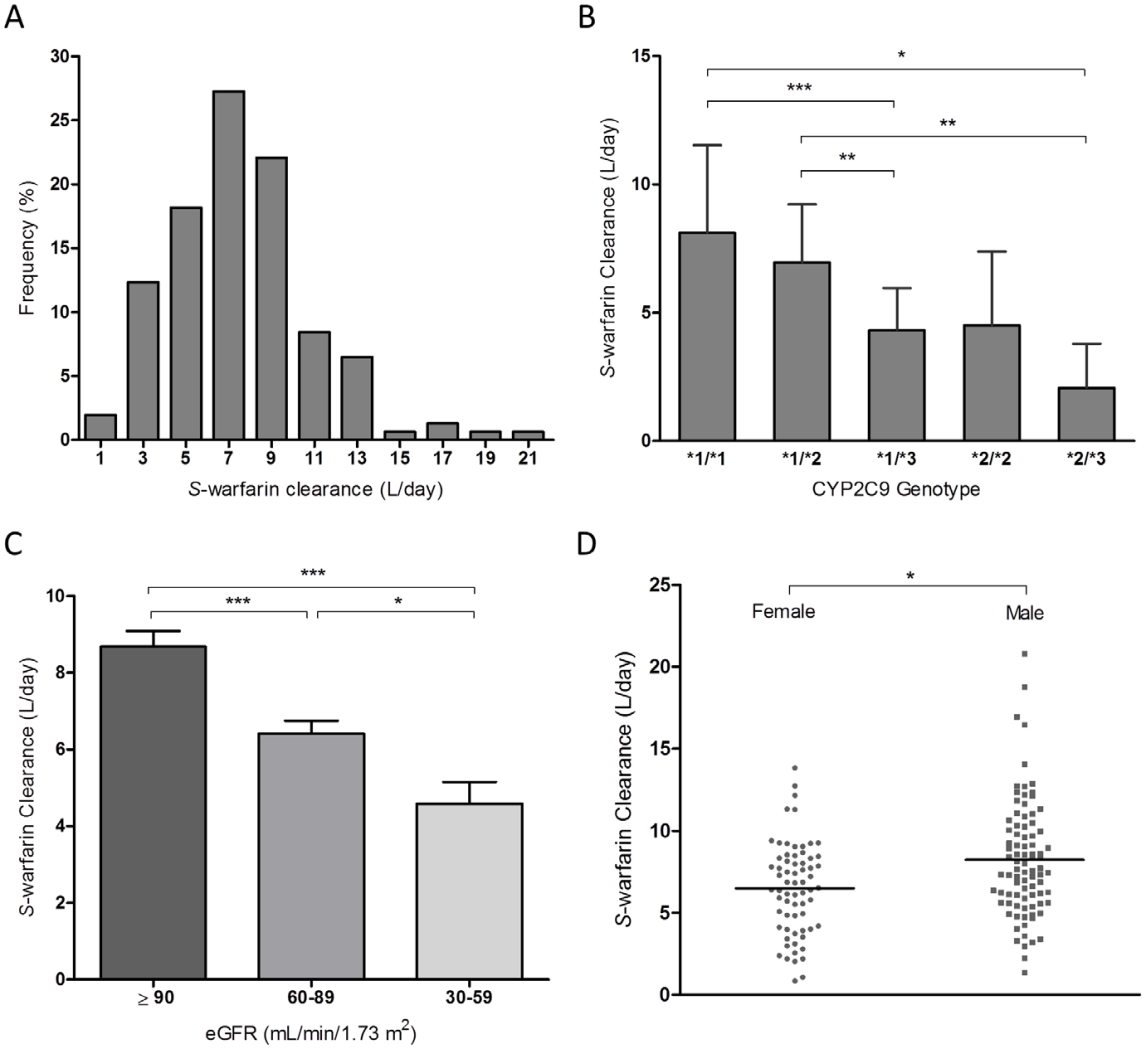
Determinants of *S*-warfarin clearance. (A) Frequency distribution of estimated *S*-warfarin clearance, shown as percent of total patients for each bin. (B) Relationship between *CYP2C9* genotype and *S*-warfarin clearance. Lines represent mean clearance. (C) *S*-warfarin clearance is significantly correlated with kidney function, as defined by eGFR. (D) Observed *S*-warfarin clearance segregated by gender. Lines represent mean clearance. eGFR, estimated glomerular filtration rate. ^*^ P<0.05, ^**^ P<0.005, ^***^P<0.0005

Mean *S*-warfarin clearance was 7.5 L/day (SD 3.4) with a range of 0.8 to 20.8, indicating a more than 20-fold interindividual variability in *S*-warfarin PK (Figure 2A). *S*-warfarin clearance was significantly associated with *CYP2C9* genotype with mean clearance values of 8.1, 7.0, 4.3, 4.5, and 2 L/day, for *CYP2C9* ^*^1/^*^1, ^*^1/^*^2, ^*^1/^*^3, ^*^2/^*^2, and ^*^2/^*^3 genotypes, respectively (Figure 2B). Interestingly, lower *S*-warfarin clearance was observed in patients with decreased renal function as estimated by glomerular filtration rate (eGFR) (Figure 2C, P<0.0001). The cohort average eGFR was 91 mL/min/1.73 m^2^ (SD 23) with a range of 22 to 140. Moreover, eGFR was significantly decreased with increase in age (P<0.0001, data not shown). Gender also had an influence on *S*-warfarin clearance, where on average, females had significantly lower clearance compared to males (Figure 2D, P<0.001). With stepwise regression, clearance was found to be dependent on *CYP2C9* ^*^3 allele, kidney function, gender, and *CYP2C9* ^*^2 allele, in order of covariate entry into the regression equation. *VKORC1* genotype was without influence on *S*-warfarin clearance. The r^2^ of the final model for clearance estimation was 36.5%. Parameter estimates of the final clearance model and bootstrap validation results are given in Table 1.

**Table 1:** Multiple linear regression analysis of independent predictors of *S*-warfarin clearance (L/day).

Entry into model	Predictor Variable	B	Standard error	95% CI	R^2^ after entry (%)	P in final model
-	Intercept	3.105	0.072	2.966, 3.248	-	<0.0001
1	*CYP2C9* ^*^3, per allele	−0.812	0.151	−1.093, −0.510	14.3	<0.0001
2	eGFR^a^	−0.278	0.080	−0.417, −0.145	24.9	<0.0001
3	Gender (F)	−0.357	0.081	−0.511, −0.186	32.3	<0.0001
4	*CYP2C9* ^*^2, per allele	−0.274	0.080	−0.427, −0.113	36.5	<0.0001

CI, confidence interval; *CYP2C9*, cytochrome P450 2C9; eGFR, estimated glomerular filtration rate in mL/min/1.73m^2^; F, female.

a Coded as follows: ≥90 mL/min/1.73 m^2^, 0; 60–89, 1; 30–59, 2; 15–29, 3; ≤15, 4.

### Therapeutic *S*-warfarin plasma concentration correlates with *VKORC1* genotype

**Figure 3:**
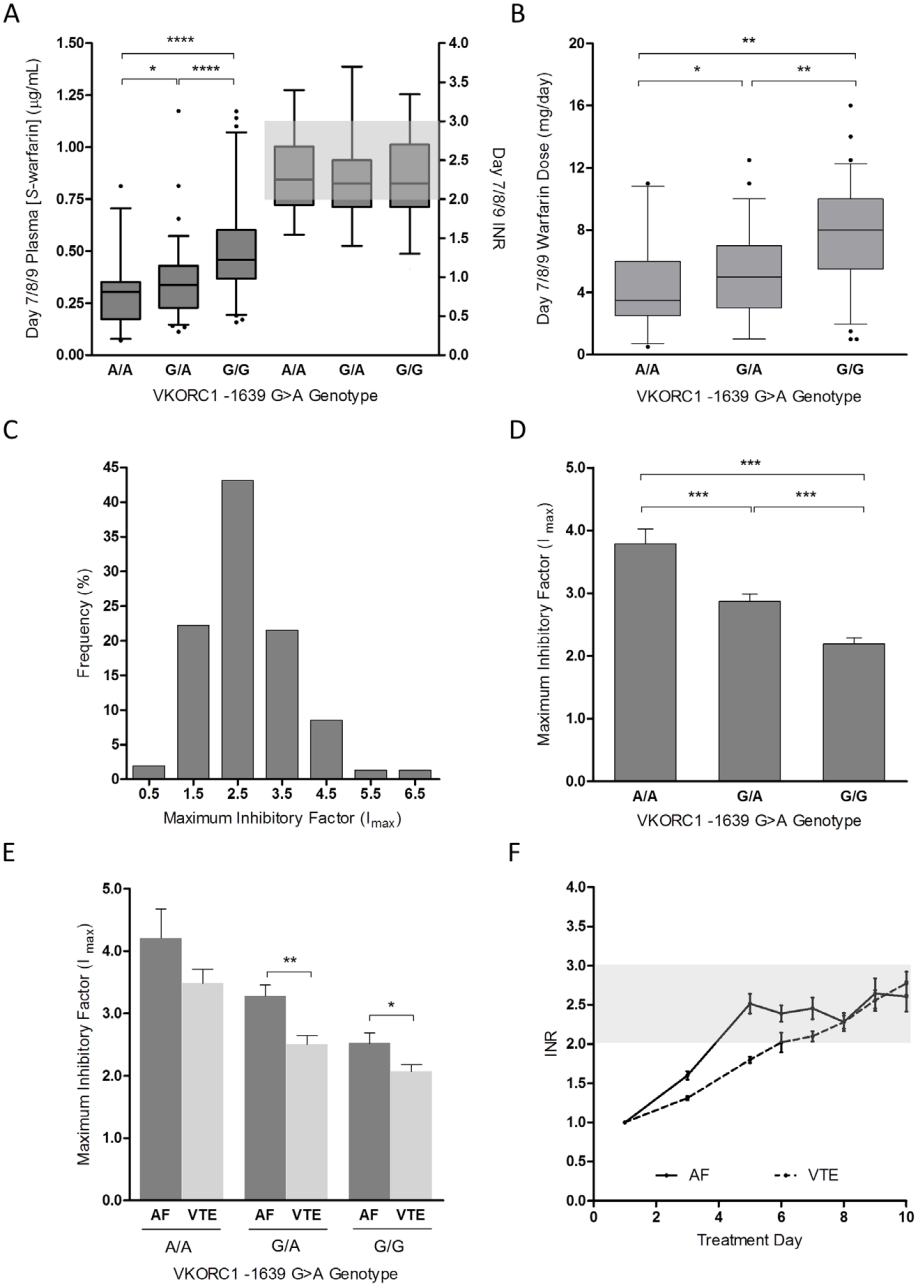
Determinants of maximal inhibitory factor, I_max_. (A) Box-and-whisker plots of *S*-warfarin plasma concentration and INR on days 7/8/9 segregated by *VKORC1* -1639G>A genotype. Box-and-whisker plots representing *VKORC1* gene-dose effect during initiation. The top and bottom of the boxes represents 25^th^ and 75^th^ percentile, respectively; median is represented by the middle line, whiskers are the 95% CI, and outliers are identified as closed circles. (B) Warfarin daily dose on days 7/8/9 with respect to *VKORC1* genotype. (C) Frequency distribution of estimated I_max_, shown as percent of total patients for each bin. (D) Association between *VKORC1* genotype and I_max_. Results are represented as mean with standard deviation. (E) Additive effect of indication for warfarin therapy and *VKORC1* genotype on I_max_. (F) INR time course for patients with AF and VTE over the initial 10 days of therapy with common genetics-guided dosing protocol. Results are represented as mean with 95% CI of the standard error. AF, atrial fibrillation; INR, international normalized ratio; VTE, venous thromboembolism. ^*^ P<0.05, ^**^ P<0.01, ^***^ P<0.001, ^****^ P<0.0001.

*S*-warfarin plasma concentrations on day 7/8/9 were statistically different between *VKORC1* -1639G>A genotype groups (P<0.0001) despite similar INR (Figure 3A). Patients carrying at least one -1639G>A allele required lower plasma concentrations than wild-type patients for similar therapeutic efficacy, and this was gene-dose dependent (Figure 3A, 3B). The mean plasma *S*-warfarin concentrations for *VKORC1* A/A, G/A, and G/G genotype groups were 0.291 ng/mL (SD 0.157), 0.347 ng/mL (0.170), and 0.503 ng/mL (0.217), corresponding to mean warfarin daily doses of 4.4 mg (2.7), 5.0 mg (2.5) and 7.6 mg (3.0), respectively.

### Determinants of *S-*warfarin PD

The mean I_max_ value for subjects was 2.7 (SD 1.0), with a range of 0.3 to 6.9, demonstrating a more than 20-fold interindividual variability in *S*-warfarin PD (Figure 3C). A significant relationship between *VKORC1* -1639G>A genotype and I_max_ (Figure 3D, P<0.0001) was observed. The mean I_max_ values for *VKORC1* A/A, G/A, and G/G genotypes were 3.7 (SD 1.2), 2.8 (1.0) and 2.2 (0.7), respectively.

Stepwise regression analysis indicated that I_max_ was dependent on *VKORC1* genotype, indication for warfarin, pre-treatment plasma proteins induced by vitamin K absence (PIVKA-II) concentration, *CYP4F2* 1297C>T genotype, and weight, in order of covariate entry into the regression equation. The r^2^ of the final model for I_max_ estimation was 41%. Parameter estimates of the final I_max_ model and the bootstrap validation results are given in Table 2. The mean baseline PIVKA-II concentration was 7.1 ng/mL (SD 4.8), with a range of 1.8 to 30.6, indicating that majority of our patients did not exhibit vitamin K deficiency. I_max_ was greater in patients with AF than VTE, denoting that VTE patients were more resistant to warfarin's therapeutic effect. Moreover, there was an additive effect of *VKORC1* genotype and indication, where VTE patients had lower I_max_ than AF patients irrespective of *VKORC1* genotype (Figure 3E). VTE patients who were *VKORC1* G/G carriers had the lowest average I_max_ (2.1) while AF and A/A carriers had the highest I_max_ (4.4). These findings imply that differences in INR response would be evident between patients with AF and VTE when warfarin is initiated by a common dosing protocol. Indeed, we found a more rapid response in patients with AF in comparison to VTE over the first week of therapy (Figure 3F), despite that the WRAPID protocol eliminated the previously known genetic and clinical contributors to early response variability.[29]

**Table 2:** Multiple linear regression analysis of independent predictors of I_max_.

Entry into model	Predictor Variable	B	Standard error	95% CI	R^2^ after entry (%)	P in final model
-	Intercept	1.383	0.101	1.156, 1.570	-	<0.0001
1	*VKORC1*, per allele	0.211	0.030	0.148, 0.267	26.3	<0.0001
2	Indication (VTE)	−0.281	0.049	−0.380, −0.190	34.9	<0.0001
3	PIVKA-II (ng/mL)	0.017	0.007	0.005, 0.033	37.9	<0.05
4	*CYP4F2*, per allele	−0.072	0.031	−0.133, −0.009	39.6	<0.05
5	Weight (Kg)	0.002	0.001	0.000, 0.004	41.0	<0.05

CI, confidence interval; *CYP4F2*, cytochrome P450 4F2; I_max_, maximal inhibitory factor; PIVKA-II, proteins induced by vitamin K absence; *VKORC1*, vitamin K epoxide reductase complex subunit 1; VTE, venous thromboembolism.

### Correlation of *VKORC1* genotype to hepatic VKOR protein levels

**Figure 4:**
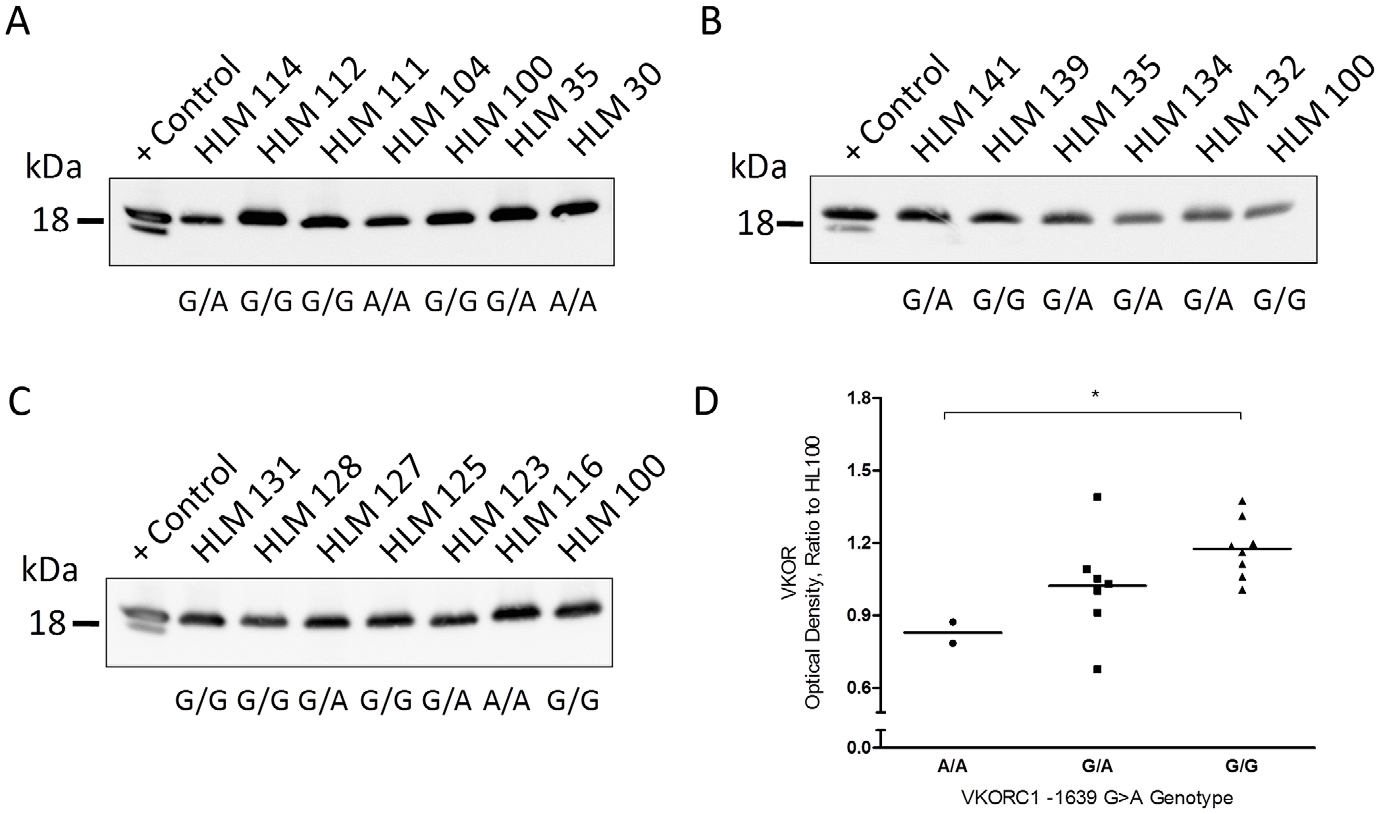
The influence of *VKORC1* -1639G>A promoter genotype on hepatic VKOR protein expression levels. (A, B, C) VKOR expression determined in 17 healthy human livers by Western blot analysis. The band intensity was normalized to HLM100. A positive control sample was included on each blot. (D) Semiquantitative measurement of hepatic expression in relation to *VKORC1* genotype. ^*^ P<0.01

With Western blot analysis, hepatic microsomal VKOR had electrophoretic mobility consistent with an 18 kDa protein while the two immunoreactive bands observed in over-expressed VKOR control samples likely represent differentially glycosylated forms of the protein (Figure 4A, 4B, 4C). VKOR protein level was significantly correlated to *VKORC1* genotype (Figure 4D, P<0.05), where the *VKORC1* G allele was associated with higher liver enzyme level than the A allele.

### Simulated anticoagulation response with different warfarin initiation protocols

**Figure 5:**
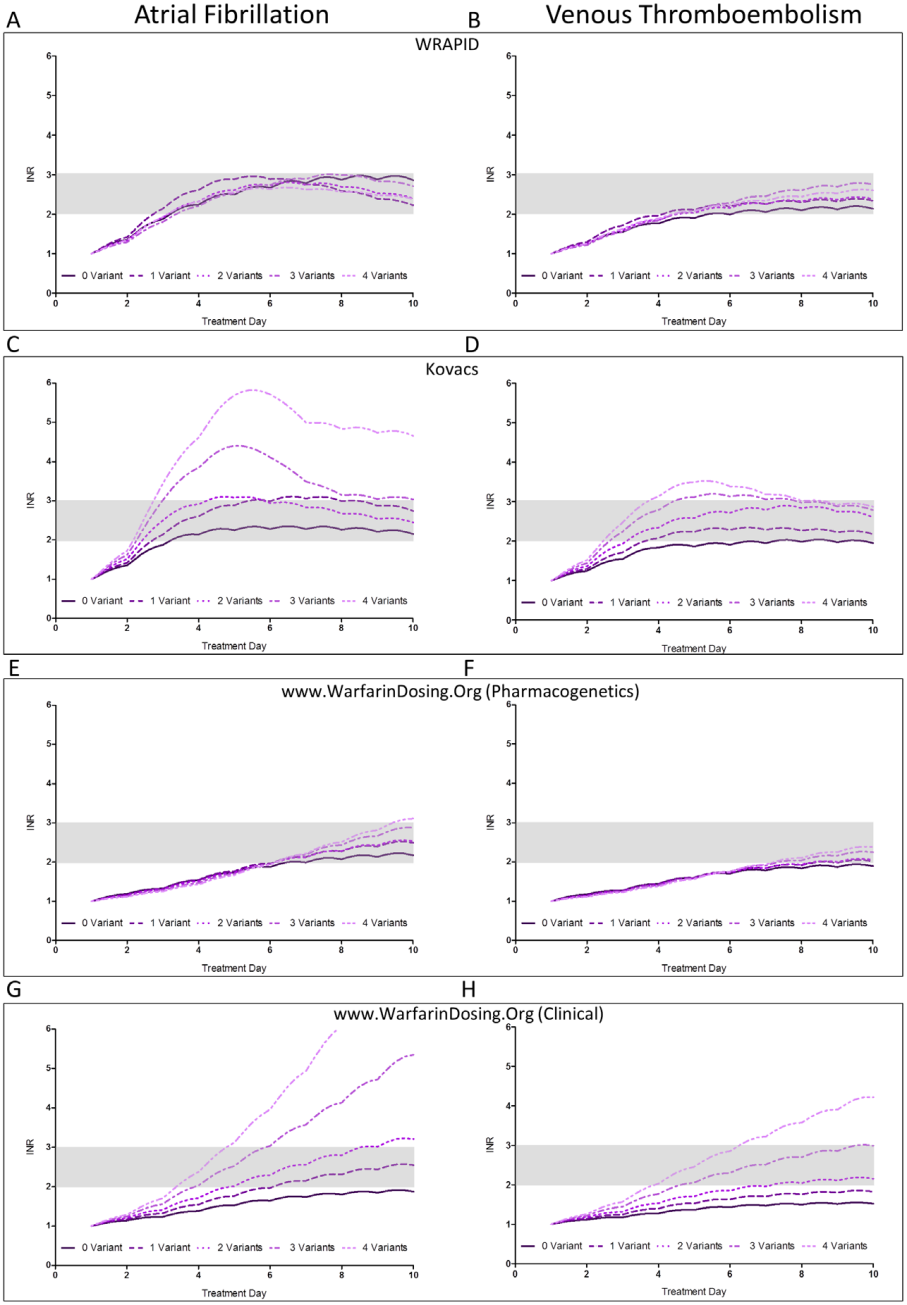
Model predicted response curves following warfarin initiation using various initiation protocols. Simulations were performed using non-genetics and genetics-based nomograms for typical AF and VTE patients harbouring variable number of variant alleles. The genotype of zero-variant patients is *VKORC1*G/G-*CYP2C9* ^*^1/^*^1. Patients carrying 1 variant allele have one of the following genotype combinations: *VKORC1*G/A-*CYP2C9* ^*^1/^*^1, *VKORC1*G/G-*CYP2C9* ^*^1/^*^2, or *VKORC1*G/G-*CYP2C9* ^*^1/^*^3. Patients carrying 2 variant alleles have one of the following genotype combinations: *VKORC1*A/A-*CYP2C9* ^*^1/^*^1, *VKORC1*G/A-*CYP2C9* ^*^1/^*^2, *VKORC1*G/A-*CYP2C9* ^*^1/^*^3, or *VKORC1*G/G-*CYP2C9* ^*^2/^*^2. Patients carrying 3 variant alleles have one of the following genotype combinations: *VKORC1*A/A-*CYP2C9* ^*^1/^*^2, *VKORC1*A/A-*CYP2C9* ^*^1/^*^3, *VKORC1*G/A-*CYP2C9* ^*^2/^*^2, or *VKORC1*G/A-*CYP2C9* ^*^2/^*^3. Patients carrying 4 variant alleles have one of the following genotype combinations: *VKORC1*A/A-*CYP2C9* ^*^2/^*^2, or *VKORC1*A/A-*CYP2C9* ^*^2/^*^3. AF, atrial fibrillation; VTE, venous thromboembolism.

To demonstrate the utility of the PK-PD model, we simulated and compared INR response profiles of individuals with different combinations of covariates using different published dose initiation schemes.[25], [29], [40], [41], [42] Specifically, we compared response-time curves of typical patients following initiation with our WRAPID protocol,[29] the Kovacs nomogram (non-pharmacogenetics, validated in VTE) with the day 8 dose refinement algorithm,[42] and finally, initiation with the pharmacogenetics-based as well as clinical-only maintenance dose algorithms available at www.warfarindosing.org incorporating the recently published day 4 dose refinement algorithm[25], [40]. Doses were adjusted according to simulated INR values on days 3, 5, and 8 for WRAPID and Kovacs and on day 4 for warfarindosing.org as per nomogram. Clearance and I_max_ values were calculated based on regression equations (Tables 1 and 2) for various *VKORC1* and *CYP2C9* genotype combinations in typical AF and VTE patients. Homozygous *CYP2C9* ^*^3 patients were not considered in the simulations as we did not encounter such individuals in our population. We used *CYP4F2* wild-type C/C genotype (increased sensitivity) for all calculations of I_max_. Figure 5 illustrates the predicted effect of *VKORC1* or *CYP2C9* variant allele burden on responses of AF and VTE patients initiated with WRAPID nomogram (5A, 5B), Kovacs nomogram (5C, 5D), warfarindosing.org genetics (5E, 4F), and clinical nomogram (5G, 5H), respectively. The simulated response curves indicate that increased possession of variant alleles is associated with slightly greater time above therapeutic INR with fixed 10 mg loading doses and iterative response-based Kovacs nomogram than initiation strategies which incorporate genetic and patient factors. In contrast, pharmacogenetics-guided initiation schemes eliminated the genotype-dependent response differences. Furthermore, pharmacogenetics-guided dosing nomograms resulted in comparable rise to optimal anticoagulation response among different genotypes within groups of AF and VTE patients. Evidently, loading dose was not used in simulations of patients initiated with warfarindosing.org and thus, the time to reach optimal anticoagulation was approximately 3 days slower as compared to the WRAPID nomogram. Simulations with the warfarindosing.org clinical algorithm indicate that there would be significant differences in initial INR responses as the burden of genetic variants increases.

## Discussion

Warfarin initiation is a challenging therapeutic phase, associated with the highest occurrence of major bleeding events and thromboembolism.[43], [44], [45], [46] Thus, effective initiation protocols that pre-emptively account and adjust for interindividual variability have significant potential to improve warfarin anticoagulation therapy.

A major contributor to dose requirement and response is *S*-warfarin clearance. The analysis demonstrates that kidney function, gender, *CYP2C9* ^*^2 and ^*^3 genotype are major determinants of *S*-warfarin clearance. The finding that *S*-warfarin clearance is reduced in renal impairment supports recent studies that found relationships between both warfarin dose requirement and propensity for over-anticoagulation with kidney function.[47], [48] Although age has been correlated with decreased warfarin clearance, we failed to observe this relationship after multivariate regression that included both age and eGFR.[7], [8], [9], [27] It is plausible that age, as a contributor to clearance, somewhat reflects age-related decline in renal function. Indeed, we note that including eGFR as an additional factor into the regression analysis resulted in 36.5% of clearance variation explained, while only 27.6% of this variation was accounted for when eGFR was absent and age included in the analysis. Interestingly, gender was a significant independent contributor to *S*-warfarin clearance in this study, with females having 22% lower *S*-warfarin clearance than males. While females require lower doses than males for similar anticoagulation quality and efficacy [49], there remain conflicting reports on the role of gender on *S*-warfarin pharmacokinetics. [7], [8], [27] Drug interactions, particularly with amiodarone and antifungals, are significant contributors to variable warfarin response.[50], [51], [52] Because of the limited number of patients in this cohort taking these medications, we did not find associations between concomitant drugs and warfarin clearance. Larger studies in patients are required to better characterize the quantitative influence of co-administered drugs on warfarin clearance.

While determinants of *S*-warfarin PK have been studied, less is known regarding determinants of the *S*-warfarin plasma concentration-response relationship. We identified *VKORC1*, weight, indication for warfarin, PIVKA-II and *CYP4F2* genotype as significant predictors of *S*-warfarin I_max_, the PD parameter that governs the magnitude of observed anticoagulation INR response. In addition, we demonstrate that promoter -1639G>A SNP results in lower hepatic VKOR protein expression. In concordance with that previously observed for warfarin-stabilized patients,[53] there was a significant relationship between *VKORC1* genotype and *S*-warfarin plasma concentrations at the end of the initiation phase where therapeutic INR was achieved. Taken together, the *VKORC1* -1639G>A promoter SNP confers lower hepatic expression, thus lower plasma *S*-warfarin concentrations and dose required for optimal anticoagulation.

The finding that I_max_ differences exist between AF and VTE patients, following adjustment of confounding variables such as age and weight, suggests that indication for treatment maybe a prominent contributor to response variability during initiation. This may be attributed to different coaguability states among patients during therapy initiation, in addition to inherent disease differences between the two subsets of patients. Further studies are required to investigate the physiological basis mediating the PD differences between AF and VTE patients. It is also of interest to know whether this dynamic difference would diminish or be maintained throughout the course of anticoagulation therapy. Indeed, indication for anticoagulation was an independent determinant of maintenance dose in the present cohort and one dose algorithm (warfarindosing.org) incorporates VTE as a factor requiring higher warfarin maintenance dose.[25] In the present study population, we find a 2.3 mg/day difference in mean maintenance dose between AF and VTE patients. This value is greater than the 0.7 mg/day difference predicted by the WRAPID maintenance dose algorithm when accounting for the average age (21 yrs) and weight (6.5 kg) differences between the AF and VTE groups. While a component of this maintenance dose difference is likely related to the lack of consideration of renal function differences among the disease groups with the WRAPID algorithm, the current PK-PD analysis suggests that indication for anticoagulation remains a contributor to warfarin dose.

In agreement with previous studies linking *CYP4F2* genotype and vitamin K intake to warfarin dose requirement,[18], [23], [24] our data demonstrate that pre-treatment plasma PIVKA-II levels and *CYP4F2* 1297G>A genotype affects *S*-warfarin PD sensitivity during initiation. PIVKA-II, a des-carboxylated form of prothrombin, is a direct biomarker for liver vitamin K status and dietary intake.[54] We did not find a relationship between PIVKA-II level and *CYP4F2* genotype during statistical analysis or collinearity during stepwise regression of I_max_ for this cohort of patients. Taken together, our findings highlight the importance of the balance between the VKOR agonist (vitamin K) and antagonistic (warfarin) concentrations in achievement and maintenance of optimal anticoagulation, particularly during initiation.

An important outcome of this study was the formulation of an overall PK-PD model that incorporates the determinants of warfarin kinetics and response. We demonstrate the utility of the model to predict *S*-warfarin concentrations and INR response curves in simulated individuals initiated with different protocols. The simulations predict substantial differences in initial anticoagulation responses depending on the initiation scheme, indication for warfarin treatment and burden of genetic variation in *CYP2C9* and *VKORC1*. Within all of the initiation protocols examined, AF patients would be predicted to have greater initial response than VTE. The indication difference exaggerates the effect of genetic polymorphisms on response especially for initiation protocols that do not consider *CYP2C9* and *VKORC1* genotypes. Interestingly, the simulations indicate that the Kovacs nomogram results in a safe and rapid initiation in VTE patients, consistent with the observed good safety profile in real-world patients.[41], [55] On the other hand, the Kovacs nomogram is predicted to be less optimal for in AF as it may pose an over-anticoagulation risk in these patients. Simulations in patients initiated with pharmacogenetics-based dosing algorithms suggest they would be safe and effective for both patient populations and that the response curves of individuals possessing variant alleles were similar. In comparing initiation with WRAPID and warfarindosing.org, time to therapeutic range was delayed without the use of loading dose. In the case for VTE, delayed attainment of therapeutic INR may have economic consequences as bridging therapy with low molecular weight heparins would need to be extended when loading doses are not administered. The simulations also forecast the time-course of initial INR responses in the large multi-centered randomized clinical trial comparing outcomes between pharmacogenetic and clinical-based warfarin dosing (Clarification of Optimal Anticoagulation Through Genetics, COAG trial) as defined by warfarindosing.org algorithms. The model predicts significant differences in INR response between the two dosing methods during initiation, particularly for patients harbouring variant alleles.

While maintenance dose prediction algorithms typically utilize a number of clinical and genetic parameters, these models are not designed to delineate how each parameter affects warfarin PK, PD or both. The formal PK-PD analysis described herein demonstrates that the interindividual variation in both components of the overall warfarin response can be separated and quantitatively ascribed to respective combinations of non-genetic and genetic factors. Moreover, our integrated PK-PD model allows for robust prediction of INR response profiles particularly during initiation phase of therapy following any initiation-dose scheme, in addition to assessment of covariate effect on responses by altering PK or PD estimates. It should be noted that based on the current model form and input parameters, the PK determinants only account for 36.5% of the variability observed in *S*-warfarin clearance, while PD determinants accounted for 41% of the I_max_ variation. For this reason, it is expected that the current model would not provide precise response estimations on an individual patient basis due to the large variation still unaccounted for.

In conclusion, the data presented here provides additional insight into the combination of patient characteristics contributing to warfarin PK and PD variability, in turn allowing better prediction of anticoagulation response outcomes without the need for intensive sampling of drug concentrations. Until there is a better understanding of additional determinants of PK and PD variation, and better quantitative characterization of drug-drug interactions, the current model may be useful for predicting outcomes in populations of patients within the context of comparing the effectiveness of various dosing algorithms in early attainment and maintenance of therapeutic INR responses and in guiding future study designs.
